# Pregnancy With Diabetic Ketoacidosis and Hypertriglyceridemia-Induced Acute Pancreatitis: The Enigmatic Triad

**DOI:** 10.7759/cureus.50862

**Published:** 2023-12-20

**Authors:** Nibedita Mishra

**Affiliations:** 1 Medicine, Tata Main Hospital, Jamshedpur, IND

**Keywords:** diabetic ketoacidosis, cholelithiasis, pregnancy, hypertriglyceridemia, pancreatitis

## Abstract

Acute pancreatitis in pregnancy is a rare condition. Acute pancreatitis due to hypertriglyceridemia in a case of diabetic ketoacidosis is also a very rare condition with a very high mortality rate. Here, we report a case of a 34-week pregnancy with pregnancy-induced hypertension, diabetic ketoacidosis, and hypertriglyceridemia-induced acute pancreatitis with underlying gallstone disease, which might have contributed to the etiology of pancreatitis. There is no report of such a case in the available literature. The patient underwent a lower segment cesarean section and was conservatively treated for all other comorbidities and had a successful outcome.

## Introduction

In a pregnant female patient, acute pancreatitis (AP) can present as abdominal pain relatively rarely. Its incidence is 1/1000-10,000 depending on the diagnostic criteria used [1]. Pancreatitis occurring in pregnancy can also occur when associated with other conditions like gallstone disease, hypertriglyceridemia (HTG), and abuse of alcohol [2]. It appears that HTG is responsible for worsening the severity score and prognosis of disease [3]. Triad of diabetic ketoacidosis (DKA), AP, and HTG is seen as a rare combination, and a high mortality rate of up to 80% has been reported in such conditions. This has been referred to as "the enigmatic triangle." It is said to occur in 4% of DKA cases [4]. There is no report of a concurrent diagnosis of DKA with HTG in a case of pregnancy causing AP. We report such a case here who in addition to the above also had gestational hypertension and ultrasonographic features of cholelithiasis and acute cholecystitis, which also could have contributed to the occurrence of pancreatitis.

## Case presentation

**Figure 1:**
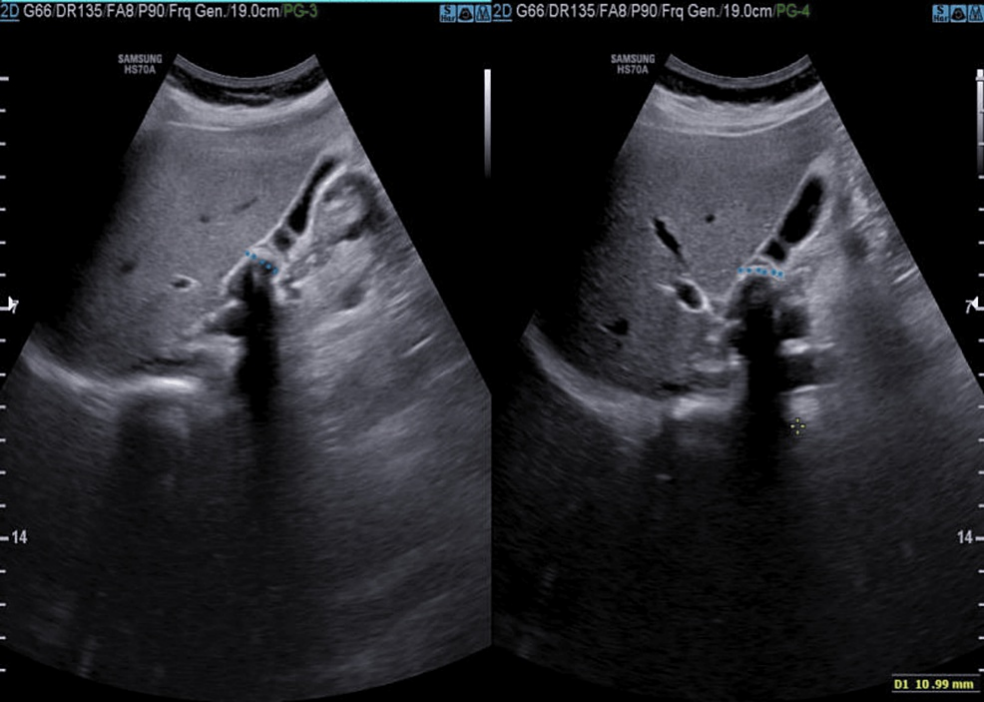
Ultrasound of the abdomen Gall bladder partially contracted with thick and edematous wall and showing 1.4 cm calculus impacted at the neck.

A 30-year-old primigravida at 34 weeks of gestation, with gestational hypertension (GHTN), was admitted to the hospital with abdomen pain and less fetal movement. She was receiving labetalol 100 mg twice daily for hypertension detected two weeks before hospitalization. On admission, she was found to have high blood sugar, for which she was put on metformin and Actrapid insulin on a sliding scale. Cardiotocogram (CTG) was monitored six hourly. The next morning, urine ketone was reported as 4+, for which urgent physician consultation was sought. She was started on broad-spectrum antibiotics as she developed a fever and DKA and was treated with IV fluids along with Actrapid infusion. Ultrasound of the whole abdomen revealed evidence of 1.4 cm calculus impacted at the neck of the gall bladder with features of cholecystitis (Figure 1). Significant blood reports included a raised serum amylase and lipase level, a chylous serum with a very high triglyceride level, and a glycosylated hemoglobin (HBA1C) level of 10.4%, indicating uncontrolled blood sugar. Surgical consultation was sought, which ruled out active surgical intervention. Thus, a provisional diagnosis of “34 weeks’ pregnancy with gestational hypertension and diabetes, diabetic ketoacidosis, cholelithiasis with cholecystitis, hypertriglyceridemia with acute pancreatitis” was made (Table 1). The next day, she had a spontaneous rupture of membranes. The decision for high-risk lower segment cesarean section (LSCS) was taken in view of multiple comorbidities and non-progress of labor. Blood loss was 600-700 ml, and around 200 ml of chylous peritoneal fluid was drained. Postoperatively, she was shifted to the high-dependency unit (HDU). Arterial blood gas (ABG) on admission to HDU showed severe metabolic acidosis with a pH of 7.1, serum lactate of 2.6, and bicarbonate (HCO3) level of 13.3 meq/l. She was put on elective ventilation with sedation and treated with IV antibiotics, IV metronidazole, IV fluids as per inferior vena cava (IVC) monitoring, and Actrapid infusion for ketosis. The next day, she was off sedation and put on a weaning mode of ventilation. Her ABG parameters had improved, and serum amylase showed a downward trend. After a T-piece trial, she was extubated, and intermittent non-invasive ventilation (NIV) support was advised. After two days, she was shifted to the gynecology postoperative ward and a similar line of management continued. In view of cholelithiasis and pancreatitis along with diabetes, surgical and medical review was sought. Since the active surgical intervention was ruled out, she was shifted to the medical ward for further management. Her amylase levels gradually came down and antihyperlipidemic drugs started. Blood pressure and sugar were controlled with antihypertensive drugs and insulin. On the 8th day of LSCS, she was discharged on oral antibiotics and other supportive treatment.

**Table 1:** Serial investigation reports g: gram; dL: deciliter; cmm: cubic millimeter; AST: aspartate aminotransferase; ALT: alanine aminotransferase; ALP: alkaline phosphatase; U/L: units per liter; S.: serum; PT: prothrombin time; INR: international normalized ratio; B: blood; HIV: human immunodeficiency virus; HCV: Ab: hepatitis C virus antibody; HBs Ag: Australia antigen; R/E: routine examination; HBA1C: glycosylated hemoglobin; HDL: high-density lipoprotein; Na: sodium; K: potassium; meq/L: milliequivalent per liter; CPK: creatine phosphokinase; CKMB: creatine kinase myoglobin binding; IU/L: international unit per liter.

Parameters	Day 1	Day 2	Day 3	Day 4	Day 5	Day 7	Day 8
Hemoglobin (g/dL)		10.8			10.6	10.9	10.3
Total leucocyte count/cmm	14000				12600	14400	12700
Differential count	N66, L26, E2, M6	N76, L16, M7, B1			N75, L16, M8	N74, M8, L16	N74, L16
Platelet count/cmm	130000	115000				187000	231000
S. bilirubin (mg/dL)		0.5					
AST (U/L)	10.0	21.1					
ALT (U/L)	29.2	10.1					
ALP (U/L)	142	136.3					
S. protein (mg/dL)	6.72	5.6					
Albumin (mg/dL)	3.06	2.51					
Globulin (mg/dL)	3.66	3.12					
PT INR	0.88		1.36				
B urea (mg/dL)	14.0	15					
S. creatinine (mg/dL)	0.48	0.59			0.55		
S. lipase	393.4	243.6			215	295	
S. amylase	136.3				65	72	
S. HIV	Negative						
HCV Ab	Negative						
HBS Ag	Negative						
Urine R/E	Glucose - 250 mg/dL, Protein - negative, Acetone - 1+		Glucose - 100 mg/dL, Protein - 236 mg/dL, RBC -plenty				
Urine acetone	1+	4+	1+				
HBA1C (%)	10.4						
Serum cholesterol(mg/dL)		823	481				
Serum triglyceride (mg/dL)		Chylous serum	586				
S. HDL (mg/dL)		151					
Serum Na (meq/L)	123	124	127	132	135	135	
Serum K (meq/L)	3.7	3.8	3.7	3.9	3.5	4.1	
Serum chloride (meq/L)	91	94	102	102	102	103	
Widal test	Negative						
CPK (IU/L)		105.4					
CKMB (IU/L)		69.3					
S. calcium (mg/dL)		8.01					
S. phosphorus (mg/dL)		2.22					
S. magnesium (mg/dL)		1.64					
C-reactive protein (mg/dL)		21.67					
Chikungunya antibody		Negative					
S. fibrinogen (mg/dL)		280					

On follow-up after 11 months, it was found that her hypertension had resolved, she continues to have mild diabetes well managed with diet control and she has undergone a successful cholecystectomy (Table 2). She continues to have dyslipidemia, albeit to a lesser extent for which she is not taking any drugs. She was advised regular monitoring and treatment of her dyslipidemia to prevent further complications.

**Table 2:** Follow-up investigation reports TLC: total leucocyte count; DC: differential count; ESR: erythrocyte sedimentation rate; AST: aspartate aminotransferase; ALT: alanine aminotransferase; S.: serum; HBA1C: glycosylated hemoglobin; HDL: high-density lipoprotein; LDL: low-density lipoprotein; TSH: thyroid-stimulating hormone; FBS: fasting blood sugar.

19/08/2023 (11 months after admission)	
Hemoglobin	12.4 gm/dL
TLC	9500/cmm
DC	N 66, L 29, E 4, M 1
ESR	48 mm/1^st^ hour
S. creatinine	0.8 mg/dL
S. uric acid	4.9 mg/dL
S. cholesterol	227.5 mg/dL
S. triglyceride	305 mg/dL
LDL	129.78 mg/dL
HDL	36.6 mg/dL
S. bilirubin	0.5 mg/dL
ALT	53.5 U/L
AST	43.7 U/L
TSH	1.34 µIU/ml
Urine sugar	Negative
Urine protein	Negative
HBA1C	5.8%
FBS	146 mg/dL

## Discussion

Most of the cases of AP in pregnancy are seen either in the third trimester or in the early postpartum stage. They are associated with a high morbidity and mortality risk for the mother and the fetus [5]. The rate of maternal death was reported to be as high as 37% and the fetal death rate was 60% in cases of pregnancy with AP [3]. AP in pregnancy is a challenge for the clinician. The rare occurrence of this condition is responsible for making the clinician less suspicious of such a possibility. There are no available obstetric guidelines presently that discuss the role of HTG causing AP in pregnancy and ways to manage it. Normal pregnancy has adaptive lipid metabolism changes. These are required to ensure the metabolic requirements of the fetus and the placenta. These changes include neoglucogenesis, higher progesterone requirement, and inhibition of lipolysis. These changes can lead to high triglyceride levels in those who have some inherent disorders of lipid metabolism. Serum triglyceride levels can normally go high in the third trimester of pregnancy, but usually less than 300 mg/dL. This level is rarely responsible for precipitating pancreatitis.

It is difficult to diagnose AP in pregnancy because there are so many other conditions that can present with abdominal pain. It can also be confused with the onset of labor pain. Conditions that can mimic the pain of pancreatitis include gall bladder pathology, peptic ulcer disease, appendicitis, pyelonephritis, and even myocardial infarction. Various complications of pregnancy can also present with abdominal pain like abruptio placentae, uterine rupture, acute fatty liver of pregnancy, etc. Usually, levels of triglyceride more than 1000 mg/dl have been attributed to cause pancreatitis. But in many cases, a lower level of triglyceride has been seen to be the only precipitating factor causing pancreatitis because the lack of food intake can cause a rapid decrease in triglyceride levels. In addition to this, sometimes serum amylase levels are normal during admission, causing further difficulty in diagnosis.

It is difficult to diagnose AP in cases of DKA. Abdominal pain can also be a presenting feature of DKA. Higher levels of serum amylase and lipase may be found in one-fourth of DKA patients without any other evidence of pancreatitis [6]. To make a specific diagnosis of AP, the serum lipase and amylase levels need to be raised three-fold. However, pancreatitis induced by HTG in the case of DKA can have normal levels of amylase/lipase due to the lipemic content of the blood, which interferes with the colorimetric assay of amylase and the presence of inhibitors of the assay in plasma [4].

DKA can either be the precipitating factor for pancreatitis or may also develop as a complication. The lack of insulin can cause increased lipolysis in cases of DKA releasing fatty acids from muscle and adipose tissue. The resulting HTG causes increased synthesis of very low-density lipoprotein (VLDL) by the liver. Blood levels of lipoprotein lipase (LPL) are low, which reduces the clearance of VLDL from blood [7]. Very high levels of triglycerides (>1000 mg/dl) may precipitate pancreatitis by releasing free fatty acids in the capillaries of the pancreas. This in turn causes tissue damage by free radicals by activating pancreatic enzymes like trypsinogen [8]. Serum triglyceride level may falsely be found low if measured after 24 hours of presentation because lack of food intake grossly reduces the level of chylomicrons in blood. In the case of HTG-induced pancreatitis, measurement of triglyceride levels done after 72 hours of presentation was found to be normal [8].

Treatment of AP induced by HTG is not much different from that due to any other cause. Lack of oral feeding, measures for pain relief, and proper fluid balance are the mainstays of treatment. Infusion of insulin also helps in reducing triglyceride levels apart from controlling sugar levels. It acts by increasing the activity of lipoprotein lipase, which is responsible for the degradation of chylomicrons and VLDL [8]. Heparin infusion has also been found to reduce triglyceride levels by stimulating the release of lipoprotein lipase from endothelium. However, it has been seen that it is followed by rebound HTG as lipoprotein lipase stores are depleted from the plasma. There are multiple case reports where plasmapheresis within 48 hours of admission has given encouraging results, but there are no guidelines for its use as routine treatment [8].

Few cases are reported in the literature that had the combination of DKA and HTG‑induced pancreatitis in a case of diabetes [9]. Mortality rates as high as 80% have been described in these cases.

## Conclusions

The combination of DKA and HTG-induced AP in a case of pregnancy has not been reported so far. In addition, our patient also had ultrasonographic findings in support of cholelithiasis and acute cholecystitis, which might have played a causal role in the pathogenesis of pancreatitis.
